# Circulating ceramides are inversely associated with cardiorespiratory fitness in participants aged 54–96 years from the Baltimore Longitudinal Study of Aging

**DOI:** 10.1111/acel.12491

**Published:** 2016-05-02

**Authors:** Elisa Fabbri, An Yang, Eleanor M. Simonsick, Chee W. Chia, Marco Zoli, Norman J. Haughey, Michelle M. Mielke, Luigi Ferrucci, Paul M. Coen

**Affiliations:** ^1^ Longitudinal Studies SectionTranslational Gerontology BranchNational Institute on AgingNational Institutes of HealthBaltimoreMD21224USA; ^2^ Department of Medical and Surgical SciencesUniversity of BolognaBolognaItaly; ^3^ Laboratory of Behavioral Neuroscience, Intramural Research ProgramNational Institute on AgingNational Institutes of HealthBaltimoreMD21224USA; ^4^ Department of NeurologyJohns Hopkins University School of MedicineBaltimoreMD21224USA; ^5^ Department of Health Science Research and NeurologyMayo ClinicRochesterMN55905USA; ^6^ Translational Research Institute for Metabolism and DiabetesFlorida HospitalOrlandoFL32804USA

**Keywords:** aging, cardiovascular fitness, ceramide, morbidity, plasma sphingolipids

## Abstract

Cardiorespiratory fitness (VO_2_ peak) declines with age and is an independent risk factor for morbidity and mortality in older adults. Identifying biomarkers of low fitness may provide insight for why some individuals experience an accelerated decline of aerobic capacity and may serve as clinically valuable prognostic indicators of cardiovascular health. We investigated the relationship between circulating ceramides and VO_2_ peak in 443 men and women (mean age of 69) enrolled in the Baltimore Longitudinal Study of Aging (BLSA). Individual species of ceramide were quantified by HPLC–tandem mass spectrometry. VO_2_ peak was measured by a graded treadmill test. We applied multiple regression models to test the associations between ceramide species and VO_2_ peak, while adjusting for age, sex, blood pressure, serum LDL, HDL, triglycerides, and other covariates. We found that higher levels of circulating C18:0, C20:0, C24:1 ceramides and C20:0 dihydroceramides were strongly associated with lower aerobic capacity (*P *< 0.001, *P *< 0.001, *P *= 0.018, and *P *< 0.001, respectively). The associations held true for both sexes (with men having a stronger association than women, *P* value for sex interaction <0.05) and were unchanged after adjusting for confounders and multiple comparison correction. Interestingly, no significant association was found for C16:0, C22:0, C24:0, C26:0, and C22:1 ceramide species, C24:0 dihydroceramide, or total ceramides. Our analysis reveals that specific long‐chain ceramides strongly associate with low cardiovascular fitness in older adults and may be implicated in the pathogenesis of low fitness with aging. Longitudinal studies are needed to further validate these associations and investigate the relationship between ceramides and health outcomes.

## Introduction

Aging is associated with a progressive loss of cardiorespiratory fitness, which in turn leads to an increased risk of morbidity and mortality (Ekelund *et al*., 1988; Blair *et al*., 1989, 1995). Cardiorespiratory fitness is defined as maximal oxygen consumption (VO_2_ peak, mL kg^−1^ min^−1^) during dynamic exercise and is typically measured during a graded exercise test (Hawkins *et al*., 2007). Using this operational definition, the decline in fitness starts around the age of thirty and continues at approximately 10% per decade. It accelerates even further toward the end of the lifespan, even in healthy persons (Fleg *et al*., 2005). Cardiorespiratory fitness is a critical determinant of physical function in older adults and an accurate indicator of cardiovascular and overall health. Thus, maintaining a good level of fitness is fundamental to delaying mobility difficulty and attaining healthy longevity.

Maximal oxygen consumption is largely explained by cardiovascular adaptations in transporting oxygen to muscle as well as mitochondrial adaptations within muscle, to meet the energy demands of physical activity. Recent evidence suggests that the capacity for vasodilatation in the peripheral vasculature also plays an important role in maximal oxygen uptake (Montero, 2015). The decline in VO_2_ peak with aging has been primarily attributed to the reductions in muscle oxygen delivery, due to decreased cardiac output, and to the reductions in skeletal muscle oxidative capacity, mainly due to the mitochondrial dysfunction (Betik & Hepple, 2008). However, there is a wide interindividual variability in the rate of decline, which is only partially explained by differences in physical activity. Thus, studies of biological correlates of physical fitness are important because they may provide insight as to why some individuals experience an accelerated decline of aerobic capacity. Further, such correlations may serve as clinically valuable prognostic indicators of cardiovascular health, morbidity, and mortality risk.

Ceramides are a ubiquitous group of lipids that consist of a sphingosine linked to a fatty acid. Ceramides are known for their structural role in plasma membranes and also as important signaling molecules involved in many essential cellular processes including inflammation, immune cell trafficking, vascular and epithelial integrity, apoptosis, autophagy, and stress responses (Maceyka & Spiegel, 2014). Their precursors, the dihydroceramides, which are transiently produced during de novo sphingolipid synthesis, are also emerging as important regulators of autophagy, hypoxia, and cellular proliferation, with biological functions that are distinct and nonoverlapping with those of the more prevalent ceramides (Siddique *et al*., 2015).

In the circulation, ceramides are transported primarily in low‐density lipoproteins (LDL) and very‐low‐density lipoproteins (VLDL) (Boon *et al*., 2013). Previous studies suggested that ceramides increase with age and are associated with accelerated aging and age‐related chronic conditions, particularly cardiovascular and metabolic diseases (Gonzalez‐Covarrubias, 2013). Treatments targeting ceramides may be potentially very effective for preventing or treating these conditions (Mielke *et al*., 2015). For example, elevated plasma ceramides cause vascular endothelial dysfunction by promoting endothelial cell growth arrest, oxidative stress, senescence and death, disrupting insulin signaling and increasing inflammation (Zhang *et al*., 2012; Symons & Abel, 2013). Perhaps through these same mechanisms, ceramides may contribute to the early stages of atherosclerosis (Ichi *et al*., 2006; Bismuth *et al*., 2008) and the accumulation of ceramide in the myocardium may cause cardiac dysfunction in obese and diabetic individuals, even in the absence of hypertension and myocardial ischemia (Park & Goldberg, 2012). It is noteworthy that Jiang and colleagues found that plasma levels of sphingolipids are an independent risk factor for coronary heart disease (Jiang *et al*., 2000). In animal models, the ceramide accumulation within the myocardium is responsible for a direct toxic effect on myocardial fibrils, cardiomyocytes apoptosis, and altered cardiac metabolism (Park & Goldberg, 2012). Furthermore, although the role of ceramides in the development of cardiac failure in humans is still largely unclear, a recent study showed that plasma ceramide levels were higher in patients with congestive heart failure (Yu *et al*., 2015) and independently correlated with mortality.

Given the evidence linking ceramide to mechanisms fundamental to cardiovascular health in cell culture and animal studies, we examined the relationships between ceramides/dihydroceramides and indicators of cardiovascular health in older adults. More specifically, we determined whether the plasma levels of individual lipid species were associated with aerobic capacity in older men and women enrolled in the Baltimore Longitudinal Study of Aging after adjusting for relevant covariates.

## Results

### Participants' characteristics

The study population consisted of 443 BLSA participants, aged 54–96 (mean age (SD): 68.9 ± 9.4), of whom 258 were men (58.2%). Table 1 summarizes the main characteristics of the whole sample and stratified by sex. Sex‐specific mean values (and standard deviations) of individual ceramide species are reported in Table S1. Participant characteristics were also examined within sex‐specific tertiles of VO_2_ peak. Individuals with lower VO_2_ peak were more likely to be older, to weigh more, to be less active, to have higher systolic blood pressure, and to be affected by diabetes compared to those with higher VO_2_ peak (Table S2).

**Table 1:** Main characteristics of the whole population and according to men and women, expressed as means (standard deviations, SD) or percentage, %

	Whole (*n *= 443)	Men (*n *= 258)	Women (*n *= 185)
VO_2_ peak, mL kg^−1^ min^−1^ (SD)	24.2 (6.8)	26.0 (7.0)	21.8 (5.6)
Age, years (SD)	68.9 (9.4)	68.7 (9.5)	69.1 (9.1)
Race (% white)	82.6	88.4	74.6
Height, cm (SD)	169.5 (10.2)	175.8 (6.9)	160.6 (6.8)
Weight, kg (SD)	75.9 (15.4)	82.6 (13.3)	66.4 (12.9)
Systolic blood pressure, mmHg (SD)	128.8 (19.1)	128.5 (18.1)	129.3 (20.5)
Diastolic blood pressure, mmHg (SD)	75.5 (12.7)	76.9 (12.6)	73.4 (12.6)
Cholesterol LDL, mg dL^−1^ (SD)	115.8 (33.9)	113.5 (32.2)	119.1 (36.0)
Cholesterol HDL, mg dL^−1^ (SD)	51.4 (14.8)	46.2 (12.2)	58.5 (15.1)
Triglycerides, mg dL^−1^ (SD)	113.0 (60.7)	118.4 (64.1)	105.6 (54.9)
Smokers (current or former), %	60.3	70.2	46.5
Diabetes, %	6.1	8.5	2.7
Prediabetes, %	31.8	40.3	20.0
Levels of physical activity, %			
Very low	6.3	5.0	8.1
Low	37.2	31.4	45.4

### Exploratory analyses

**Figure 1:**
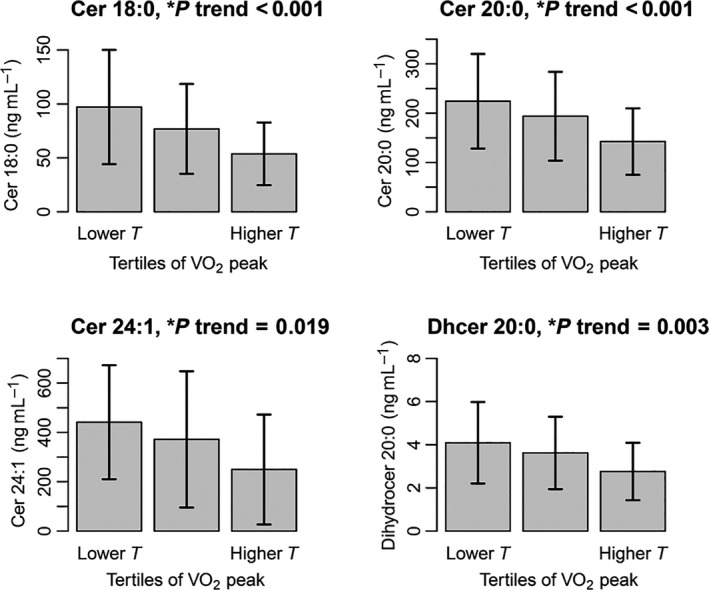
Average values of C18:0, C20:0, C24:1 ceramides and C20:0 dihydroceramide according to the tertiles of VO_2_ peak, independent of age and sex.

The crude relationship between ceramide species and VO_2_ peak was first explored in men and women, using Spearman's correlations. We found that specific ceramide (C16, C18:0, C20:0, C22:0, C24:1) and dihydroceramide species (C20:0 and C24:0) were negatively correlated with VO_2_ peak in both sexes (Table 2). For these species, graphical representations of ceramide levels and VO_2_ peak in men and in women were plotted in Figs S1 and S2, respectively. In contrast, we did not find any significant correlation between ceramide species C24:0, C26:0, C22:1, total ceramides, and VO_2_ peak (Table 2). Similar results were found when we examined the crude mean values of the ceramide species according to sex‐specific tertiles of VO_2_ peak (Fig. 1). We also explored the relationship between VO_2_ peak and sphingomyelins/dihydrosphingomyelins. However, after adjusting for potential confounders and correcting for multiple comparisons, we found no significant independent association and decided not to pursue further analyses (Table S3).

**Table 2:** Spearman's correlations between VO_2_ peak and ceramide species in men and women

	VO_2_ peak (mL kg^−1^ min^−1^)	
	Men (*n *= 258)	Women (*n *= 185)
Total ceramides (ng mL^−1^)	*r *= −0.01, *P *= 0.850	*r *= −0.11, *P *= 0.121
Ceramide 16:0 (ng mL^−1^)	*r *= −0.28, *P *< 0.001**	*r *= −0.14, *P *= 0.052*
Ceramide 18:0 (ng mL^−1^)	*r *= −0.44, *P *< 0.001**	*r *= −0.38, P = <0.001**
Ceramide 20:0 (ng mL^−1^)	*r *= −0.43, *P *< 0.001**	*r *= −0.38, *P *= <0.001**
Ceramide 22:0 (ng mL^−1^)	*r *= −0.12, *P *= 0.053*	*r *= −0.24, *P *= <0.001**
Ceramide 24:0 (ng mL)	*r *= 0.05, *P *= 0.401	*r *= −0.04, *P *= 0.594
Ceramide 26:0 (ng mL^−1^)	*r *= 0.03, *P *= 0.571	*r *= 0.09, *P *= 0.194
Ceramide 22:1 (ng mL^−1^)	*r *= −0.09, *P *= 0.157	*r *= −0.12, *P *= 0.105
Ceramide 24:1 (ng mL^−1^)	*r *= −0.39, *P *< 0.001**	*r *= −0.32, *P *< 0.001**
Dihydroceramide C20:0 (ng mL^−1^)	*r *= −0.38, *P *< 0.001**	*r *= −0.33, *P *< 0.001**
Dihydroceramide C24:0 (ng mL^−1^)	*r *= −0.17, *P *= 0.005**	*r *= −0.15, *P *= 0.043*

**P < 0.05; *P < 0.1.

### Association between ceramides and VO_2_ peak

Next, we conducted the multivariate linear regression models to test the cross‐sectional association between VO_2_ peak (mL kg^−1^ min^−1^) and ceramide species (expressed in sex‐specific standard units) while controlling for covariates. Higher levels of C18:0, C20:0, C24:1 ceramide and C20:0 dihydroceramide remained significantly associated with poorer cardiorespiratory fitness after the adjustment for age and sex (Model I, Table 3) and also for other covariates including race, height, weight, blood pressure, serum LDL, HDL, triglycerides, lipid‐lowering drug use, beta‐blocker use, diabetes status, smoking status, and the level of physical activity (Model II, Table 3). The results were substantially retained after conducting multiple comparison corrections using Bonferroni and Benjamini–Hochberg procedures. Of note, the ratio of C20:0 dihydroceramide to C20:0 ceramide was significantly and positively associated with VO_2_ peak, independent of the same covariates.

**Table 3:** Multiple linear regression models testing the association between individual molecular species of ceramide (expressed in sex‐specific standard units, SD) and VO_2_ peak (mL kg^−1^ min^−1^), after the adjustment for age and sex (Model I) and age, sex, race, height, weight, blood pressure, serum LDL, HDL, triglycerides, lipid‐lowering drug use, beta‐blocker use, diabetes status, smoke status, level of physical activity (Model II)

	Model I (age and sex adjusted)		Model II (fully adjusted†)	
	β‐coefficient (SE)	*P* values	β‐coefficient (SE)	*P* values
Total ceramides (SD)	−0.007 (0.25)	0.977	0.16 (0.24)	0.510
Ceramide 16:0 (SD)	−0.13 (0.26)	0.611	−0.34 (0.24)	0.165
Ceramide 18:0 (SD)	−1.45 (0.28)	<0.001*, #	−1.06 (0.27)	<0.001
Ceramide 20:0 (SD)	−1.37 (0.28)	<0.001*, #	−0.99 (0.26)	<0.001
Ceramide 22:0 (SD)	−0.41 (0.26)	0.112	−0.11 (0.24)	0.647
Ceramide 24:0 (SD)	0.16 (0.25)	0.524	0.29 (0.24)	0.223
Ceramide 26:0 (SD)	0.19 (0.25)	0.450	0.13 (0.24)	0.578
Ceramide 22:1 (SD)	−0.22 (0.25)	0.374	0.17 (0.23)	0.454
Ceramide 24:1 (SD)	−0.69 (0.29)	0.018#	−0.65 (0.26)	0.013
Dihydroceramide C20:0 (SD)	−1.07 (0.27)	<0.001*, #	−0.69 (0.25)	0.006
Dihydroceramide C24:0 (SD)	0.04 (0.26)	0.884	−0.04 (0.23)	0.847

^†^Covariates = age, sex, race, height, weight, blood pressure, serum LDL, HDL, triglycerides, lipid‐lowering drug use, beta‐blocker use, diabetes status, smoke status, the level of physical activity.

*P < 0.05 at Bonferroni correction; ^#^ *P *< 0.05 at Benjamini–Hochberg procedure.

### Gender differences in the strength of the association

At sex‐stratified analyses, we observed that the strength of the relationship of some species of ceramides (C16:0 and C22:0) with VO_2_ peak was different in men and women. The significance of the differential association by sex was tested by introducing a ‘sex*ceramide’ interaction term as predictor in the multiple adjusted regression model. The results of these analyses confirmed that the association of C16:0, C18:0, C20:0, C24:1 ceramides and C20:0 dihydroceramide with VO_2_ peak was significantly stronger in men than in women (P for interaction <0.05 in all cases).

## Discussion

A decline in cardiorespiratory fitness is an important and independent predictor of disability and multiple adverse health outcomes in older adults. Circulating ceramides have recently emerged as an important player in cardiovascular health through their effect on inflammation and oxidative stress. However, studies to date have not yet examined their relationship with cardiorespiratory fitness. In the present analysis, using data from participants in the BLSA aged 54 to 96 years, we found that higher levels of plasma ceramides 18:0, 20:0, 24:1 and dihydroceramides 20:0 were significantly associated with poorer aerobic capacity, even after the adjustment for potential confounders and multiple comparison correction. These results provide complementary empirical evidence to experimental studies conducted in model organisms and in humans showing that ceramides play an important role in cardiovascular health and the development of many age‐related conditions.

Our current conceptualization of VO_2_ peak as a measure of fitness in humans began almost one century ago, when the physiologist A.V. Hill postulated that the maximum oxygen uptake is limited by the ability of the cardiorespiratory system to transport oxygen to the muscles (Hill & Lupton, 1923), whose main determinants are (i) pulmonary ventilation and diffusion capacity, (ii) maximal cardiac output, (iii) oxygen‐carrying capacity, and (iv) peripheral limiting factors, including capillary density, peripheral diffusion gradients, and skeletal muscle mitochondrial capacity and efficiency. Reduced cardiac output and impaired muscular oxidative capacity are considered the principal contributors to the age‐related decline in VO_2_ peak (Betik & Hepple, 2008).

At least theoretically, high ceramide levels may have a negative impact on different steps of the pathway for the transport of oxygen from the atmosphere to the mitochondria and its utilization to produce energy. First, there is evidence that ceramides accumulate within the skeletal muscle and promote the oxidative stress and mitochondrial dysfunction (Coen & Goodpaster, 2012; Smith *et al*., 2013). Second, the accumulation of ceramide within the myocardium (lipotoxicity) has been recently proposed as a major cause of dilated cardiomyopathy in patients with diabetes and obesity and as a potential mechanism for the development of heart failure, even in the absence of hypertension and myocardial ischemia Park and Goldberg, 2012. In particular, animal studies showed that myocardial accumulation of ceramide significantly correlates with cardiac dysfunction, due to a direct toxic effect of ceramide on myocardial contractile function, the loss of cardiac myocytes via apoptosis, and altered cardiac metabolism (unbalanced glucose/fatty acid oxidation) (Zhou *et al*., 2000; Chiu *et al*., 2001; Yagyu *et al*., 2003; Park *et al*., 2007).

Ceramides are also known to negatively affect the endothelial function, which is highly correlated with aerobic capacity (Montero, 2015). In particular, ceramides trigger several pathways that induce endothelial cell death, including the activation of caspases, increasing the mitochondrial permeability and the impairment of endothelial nitric oxide synthase (eNOS) activity, with a consequent increment in vascular permeability and endothelial dysfunction (Symons & Abel, 2013). Also, ceramides have been linked to the growth arrest, cytoskeleton rearrangements, oxidative stress, and senescence of endothelial cells. Furthermore, ceramides can indirectly contribute to endothelial dysfunction by promoting insulin resistance and increasing inflammation.

Our findings are substantially in keeping with a number of previous studies in animal models and humans showing that plasma levels of ceramides are increased in obesity (Samad *et al*., 2006; Haus *et al*., 2009; Huang *et al*., 2011). In fact, obese persons frequently have lower aerobic capacity and mitochondrial dysfunction and the cause of such dysfunction is less than clear. Moreover, a recent study, including obese volunteers, endurance‐trained athletes, and individuals with type 2 diabetes, found that muscle ceramide concentration increased during acute exercise and then decreased after the recovery to levels significantly lower than their baseline values, and the authors suggested that the change in muscle ceramides may promote insulin‐sensitizing effects of acute exercise (Bergman *et al*., 2016).

Of relevance, we found that specific species of ceramides, in particular C18:0 and C20:0, show a strong association with poor aerobic capacity. Although the exact mechanisms that may link these specific lipid species to an impaired cardiovascular fitness are not fully clear, it is worth mentioning that the enzymes that are specifically involved in the synthesis of these molecules, namely ceramides synthase 1 (C18:0) and ceramides synthase 4 (C18:0 and C20:0), are primarily expressed, respectively, in the skeletal muscle and in the heart.

The mechanisms by which specific ceramide species appear to affect specific physiological function remain unclear. While there is accumulating evidence that the biological effect of ceramides is mediated by multiple mechanisms including the activation of PP2A, PKC, and NLRP3 and mitochondrial and ER stress (Chaurasia & Summers, 2015), many studies found that a few ceramides species out of a complete profile seem to play an especially relevant role. In particular, and consistent with our findings, emerging evidence in both humans and animal models shows that C18:0 and C20:0 ceramides are important in mediating negative health effects. For example, serum C16:0 and C18:0 ceramide and C18:0 sphingomyelin were recently found to be positively correlated with the markers of muscle NF‐κB activation, suggesting that these specific species could activate the intracellular inflammation (Bergman *et al*., 2015). Furthermore, in a previous study, the levels of C18:0, C20:0, C24:1, and total ceramides were found to be elevated in type 2 diabetic subjects compared to controls and were inversely correlated with insulin sensitivity, concluding that these species may contribute to insulin resistance through the activation of inflammatory mediators, such as TNF‐alpha (Haus et al., 2009). In addition, C16:0 and C18:0 ceramides have been mechanistically linked to systemic metabolic health in genetic models (Xia *et al*., 2015) and with insulin resistance in the muscle in obesity (Coen *et al*., 2010). A challenge in interpreting human profiling studies is that ceramides are biosynthetic intermediates that do not exist at a steady‐state concentration. Little is known about how they fluctuate in response to feeding or other environmental factors, and flux determinations are not typically obtained. Moreover, the subcellular location of the crucial pool of sphingolipids that regulate cell function remains unresolved. With these temporal/spatial aspects of the sphingolipidome incompletely understood, a full interpretation of these findings remains difficult.

An exploratory analysis with the sample population stratified by sex revealed that for some ceramide species (i.e., ceramides C16:0 and C22:0), but not for others (dihydroceramides C24:0), crude sex differences in the strength of their relationship to VO_2_ peak existed. Independent of potential confounders, sex differences were confirmed for C16:0, C18:0, C20:0, C24:1 ceramides and C20:0 dihydroceramide, with men showing a significantly stronger association between higher levels of ceramides and poor aerobic capacity as compared to women. This interesting finding is in keeping with previous cross‐sectional studies that reported gender differences in plasma levels of ceramides, with women generally showing higher concentrations of ceramides then men (Hammad *et al*., 2010; Bui *et al*., 2012; Ishikawa *et al*., 2013; Weir *et al*., 2013). Longitudinal sex differences in the levels of ceramides with aging were also previously described in BLSA population, with women showing a steeper trajectory of increase than men (Mielke *et al*., 2015). On the other hand, as reported by Fleg and colleagues, men tend to have higher values of VO_2_ peak, but a greater decline with aging in the levels of VO_2_ peak compared to women (Fleg *et al*., 2005). In addition, many gender‐related factors could influence the relationship between ceramides and VO_2_ peak, some of which in the current study we were not be able to fully account for, such as sex‐specific differences in lipid metabolism (Varlamov *et al*., 2015) and sex‐specific plasticity of the cardiovascular system to fitness and physical activity in older adults (Parker *et al*., 2010). Indeed, further and more specific investigations are required to fully disentangle the complex relationship between sex, ceramides, and cardiovascular health.

Our results are consistent with the findings from the preclinical literature and suggest that modulating the level of ceramides may be a new target for interventions aimed at preventing the development of cardiovascular diseases with aging. However, several limitations of the current analysis need to be addressed. First, the sample population is relatively small. Moreover, because the BLSA continuously enrolls healthy volunteers followed for life, and participation is very demanding and time‐consuming, there may be a selection bias with those enrolled in the study tending to be healthier than subjects of the same age in the overall population. Therefore, further investigations in larger and more diverse populations are required to validate our findings and confirm their generalizability. Second, because our analysis is cross‐sectional, we cannot make any temporal inference about the relationship between ceramides and VO_2_ peak. Additional longitudinal studies in older adults are required to determine whether ascertaining ceramide levels may be a clinically useful early predictor of accelerated decline in aerobic capacity. Finally, in our analysis we only measured C20:0 and C24:0 dihydroceramide species. Indeed, for the lipidomics analysis of samples from BLSA participants no other dihydroceramide species were measured. Further analyses including a full profile of dihydroceramides are required.

In conclusion, higher circulating levels of ceramides 18:0, 20:0, 24:1 and dihydroceramides 20:0 are significantly associated with lower VO_2_ peak values in older men and women, independent of potential confounders. These lipid species are likely to negatively affect both central and peripheral determinants of maximum oxygen consumption and may be important biomarkers of aerobic capacity in older adults, with a potential prognostic value. To translate these findings to the clinic, we need further studies that increase our understanding of the link between circulating ceramides and cardiovascular function and longitudinal studies that test the hypothesis that ceramides levels predict the accelerated decline of cardiovascular fitness with aging.

## Methods

### Study design and setting

The BLSA is a study of human aging established in 1958 by the National Institute on Aging Intramural Research Program. A general description of the sample and enrollment procedures and criteria has been previously reported (Stone & Norris, 1966). Briefly, the BLSA continuously enrolls healthy volunteers aged 20 and older who are followed for life with follow‐up visits conducted at intervals of 1–4 years, with a more frequent follow‐up of older persons. Participants are assessed at the National Institute on Aging Intramural Research Program Clinical Research Unit in Baltimore, Maryland, over 3 days of testing. Certified nurse practitioners and certified technicians administer all the assessments following the standardized protocols. All participants receive an extensive description of the study protocol, procedures, and the risk associated with participation and consent to be part of the study at every visit.

### Participants

The sample for the present analysis consisted of 443 BLSA participants, aged 54–96, with the data available on plasma ceramides and VO_2_ peak at the same visit. Participants self‐reported as white individuals or other racial groups. Height and weight were measured while the participants wore a gown. Body weight was measured in kilograms with a calibrated scale to the nearest 0.1 kg. Height was measured in centimeters by a stadiometer to the nearest 0.1 cm. Current and former smokers were ascertained using a self‐reported questionnaire. Medication including the use of lipid‐lowering agents and beta‐blockers was recorded by self‐report.

### Blood pressure

Systolic blood pressure and diastolic blood pressure were measured in the morning, after a light breakfast, with participants in the seated position, and following a 5‐min rest, according to a standard protocol. Blood pressure was measured three times in both arms with a mercury sphygmomanometer, and the average of the second and third measurements on both the right and the left arms was used in the present analysis.

### Fasting serum lipids

Blood samples were drawn from the antecubital vein between 7 and 8 am after an overnight fast. Plasma triglyceride and total cholesterol levels were determined by an enzymatic method (ABA‐200 ATC Biochromatic Analyzer; Abbott Laboratories, Irving, TX, USA). High‐density lipoprotein (HDL) cholesterol was measured by a dextran sulfate–magnesium precipitation procedure. Low‐density lipoprotein (LDL) cholesterol was estimated by the Friedewald formula for those participants with triglycerides no >400 mg dL^−1^.

### Diabetes status

Fasting plasma glucose was measured by the glucose oxidase method (Beckman Instruments Inc., Fullerton, CA, USA). Antidiabetic medications use was self‐reported. Diabetes was defined as fasting glucose ≥126 mg dL^−1^ and/or the use of antidiabetic drugs. Participants with fasting plasma glucose between 100 and 125 mg dL^−1^ were classified as prediabetic, otherwise as normal.

### Physical activity levels

Physical activity (PA) was ascertained in BLSA participants by a self‐reported questionnaire and quantified by assigning each activity a correspondent value in metabolic units (METS, or metabolic equivalents of resting oxygen consumption) using the coding system described by Ainsworth and colleagues (Ainsworth *et al*., 1993). The MET unit assigned to the activity was multiplied by the average number of minutes performing each activity in a 24‐h period, providing a value for PA in MET‐min day^−1^. PA levels were then categorized according to MET intensity: (i) very low (0–49 MET‐min day^−1^), (ii) low (50–249 MET‐min day^−1^), (iii) medium (250–499 MET‐min day^−1^), and (iv) high (≥500 MET‐min day^−1^).

### Cardiovascular fitness

All BLSA participants were examined by a nurse practitioner who also reviewed the exclusion criteria for graded treadmill testing (e.g., moderate aortic stenosis, unstable angina, myocardial infarction of revascularization within the last 6 months, high‐grade AV block). Oxygen consumption was measured continuously during a modified Balke protocol with the speed of the motor‐driven treadmill held constant (typically at 3.0 mph for women and 3.5 mph for men), and the grade of the treadmill progressively increased by 3% at 2‐min intervals until voluntary exhaustion. Noninvasive measurements of oxygen consumption were performed throughout the testing period. Participants exercised until volitional exhaustion, symptom onset, or technician decision to end testing. Oxygen consumption was calculated every 30 s, and the highest value was determined to be the VO_2_ peak, expressed in milliliters per kilogram per minute (mL kg^−1^ min^−1^). Gas concentrations were initially measured in BLSA by either dedicated O_2_ and CO_2_ analyzers or a medical mass spectrometer (Perkin‐Elmer MGA‐1110) (Fleg *et al*., 2005), while a MedGraphics D‐Series Gas Exchange System (Medgraphics; Medical Graphics Corporation, St Paul, MN, USA) has been introduced since 2002. Consequently, all the analyses carried out in the present study were adjusted for time of the visit (before/after 2002).

### Ceramide species

Lipid extraction of plasma was conducted using a modified Bligh and Dyer procedure (Bligh & Dyer, 1959) with ceramide C12:0 included as an internal standard (Avanti Polar Lipids, Alabaster, AL, USA). Plasma extracts were dried in a nitrogen evaporator (Organomation Associates Inc., Berlin, MA, USA) and resuspended in pure methanol just prior to analysis. An autosampler (LEAP technologies Inc., Carrboro, NC, USA) injected extracts into an HPLC (PerkinElmer, MA, USA) equipped with a reverse‐phase C18 column (Phenomenex, Torrance, CA, USA). Ceramide species were separated by gradient elution at the flow rate of 400.0 μL min^−1^. The mobile phase A consisted of 85% methanol, 15% H_2_0, and 5 mm ammonium formate. Mobile phase B consisted of 99% methanol, 1% formic acid, and 5 mm ammonium formate. Gradient conditions were as follows: a gradual increase from 100% A to 100% B over 0.5 min, hold at 100% B for 4.5 min, then a decline from 100% to 0% B during the next 1 min. Eluted sample was injected into an electrospray ion source coupled to a triple quadrupole mass spectrometer (API3000; AB Sciex Inc, Thornhill, ON, Canada). Instrument parameters were as follows: ion spray voltage (V) 5500 at a temperature of 80^◦^C with a nebulizer gas of 8 psi, curtain gas of 8 psi, and collision gas of 4 psi. The declustering potential was 80V, focusing potential 400V, entrance potential 10V, collision energy 30V, and collision cell exit potential 18V. Analysis was conducted by multiple reaction monitoring (MRM). Eight point calibration curves (0.1–1.000 ng mL^−1^) were constructed by plotting area under the curve (AUC) for each calibration standard d18:1/C16:0, d18:1/C18:0, d18:1/C20:0, d18:1/C22:0, d18:1/C24:0 normalized to the internal standard. Ceramide concentrations (ng mL^−1^) were determined by fitting the identified ceramide species to these standard curves based on acyl chain length. Instrument control and quantitation of spectral data were performed using Analyst 1.4.2 and MultiQuant software (AB Sciex Inc) (Haughey *et al*., 2004; Bandaru *et al*., 2013; Mielke *et al*., 2015).

### Statistical analysis

The cross‐sectional relationship between ceramide species and VO_2_ peak was initially explored using scatter plots and Spearman's correlations, separately in men and women. Then, because sex differences exist in both circulating levels of ceramides and VO_2_ peak values, data from the whole population were analyzed according to the sex‐specific distribution. In particular, the mean concentrations of ceramide species were calculated according to sex‐specific tertiles of VO_2_ peak and P trends estimated. Moreover, the associations between each ceramide species (after sex‐specific standardization) and VO_2_ peak (mL kg^−1 ^min^−1^) were tested using multivariate linear regression models, independent of age and sex (basic model) and other additional covariates (fully adjusted model).

In addition, adjustment for multiple testing was made by means of both Bonferroni correction and Benjamini–Hochberg procedure, which is considered more appropriate in an ‘omics’ setting than Bonferroni correction. Finally, ‘sex*ceramides’ interaction terms used to test the hypotheses that the effect of ceramides on MVO_2_ was significantly different between men and women. All analyses were performed using the SAS statistical package, version 9.3 (SAS institute Inc., Cary, NC, USA) and R 3.1.2.

## Funding

This research was supported by the Intramural Research Program of NIH, National Institute on Aging, and by a grant from the National Institutes of Health/National Institute on Aging (U01 AG37526) to MMM. Data for these analyses were obtained from the Baltimore Longitudinal Study of Aging, a study performed by the National Institute on Aging. PMC is supported by a career development award from the National Institute on Aging (K01AG044437).

## Conflict of interest

The authors have no conflict of interest to declare.

## Author contributions

Drs Fabbri and Ferrucci had full access to all the data in the study and take responsibility for the integrity of the data and the accuracy of the data analysis. Coen and Ferrucci were involved in study concept and design. Mielke, Haughey, Simonsick, Chia, and Ferrucci were involved in the acquisition of data. Fabbri, Coen, and Ferrucci analyzed and interpreted the data. Fabbri and Coen drafted the manuscript. Yang, Simonsick, Chia, Zoli, Mielke, and Ferrucci critically revised the manuscript for important intellectual content. Fabbri, Yang, and Ferrucci were involved in statistical analysis. Ferrucci supervised the study.

## Supporting information


**Fig. S1** Plots of ceramide species significantly and inversely correlated with VO_2_ peak in men.

**Fig. S2** Plots of ceramide species significantly and inversely correlated with VO_2_ peak in women.

**Table S1** Mean and standard deviation (SD) of ceramide species (ng mL^−1^) according to women and men.
**TableS2** Main characteristics of the population (mean or percentage), according to sex‐specific tertiles of VO_2_ peak, and their corresponding P trends.
**Table S3** Spearman correlations exploring the relationship between sphingomyelins and VO_2_ peak, independent of potential confounders.

## References

[acel12491-bib-0001] Ainsworth BE , Haskell WL , Leon AS , Jacobs DR Jr , Montoye HJ , Sallis JF , Paffenbarger RS Jr. (1993) Compendium of physical activities: classification of energy costs of human physical activities. Med. Sci. Sports Exerc. 25, 71–80. 8292105 10.1249/00005768-199301000-00011

[acel12491-bib-0002] Bandaru VV , Mielke MM , Sacktor N , McArthur JC , Grant I , Letendre S , Chang L , Wojna V , Pardo C , Calabresi P , Munsaka S , Haughey NJ (2013) A lipid storage‐like disorder contributes to cognitive decline in HIV‐infected subjects. Neurology 81, 1492–1499. 24027056 10.1212/WNL.0b013e3182a9565e PMC3888167

[acel12491-bib-0003] Bergman BC , Brozinick JT , Strauss A , Bacon S , Kerege A , Bui HH , Sanders P , Siddall P , Kuo MS , Perreault L (2015) Serum sphingolipids: relationships to insulin sensitivity and changes with exercise in humans. Am. J. Physiol. Endocrinol. Metab. 309, E398–E408. 26126684 10.1152/ajpendo.00134.2015 PMC4537923

[acel12491-bib-0004] Bergman BC , Brozinick JT , Strauss A , Bacon S , Kerege A , Bui HH , Sanders P , Siddall P , Wei T , Thomas MK , Kuo MS , Perreault L (2016) Muscle sphingolipids during rest and exercise: a C18:0 signature for insulin resistance in humans. Diabetologia 59, 785–798. 26739815 10.1007/s00125-015-3850-y

[acel12491-bib-0005] Betik AC , Hepple RT (2008) Determinants of VO2 max decline with aging: an integrated perspective. Appl. Physiol. Nutr. Metab. 33, 130–140. 18347663 10.1139/H07-174

[acel12491-bib-0006] Bismuth J , Lin P , Yao Q , Chen C (2008) Ceramide: a common pathway for atherosclerosis? Atherosclerosis 196, 497–504. 17963772 10.1016/j.atherosclerosis.2007.09.018 PMC2924671

[acel12491-bib-0007] Blair SN , Kohl HW 3rd , Paffenbarger RS Jr , Clark DG , Cooper KH , Gibbons LW (1989) Physical fitness and all‐cause mortality. A prospective study of healthy men and women. JAMA 262, 2395–2401. 2795824 10.1001/jama.262.17.2395

[acel12491-bib-0008] Blair SN , Kohl HW 3rd , Barlow CE , Paffenbarger RS Jr , Gibbons LW , Macera CA (1995) Changes in physical fitness and all‐cause mortality: a prospective study of healthy and unhealthy men. JAMA 273, 1093–1098. 7707596

[acel12491-bib-0009] Bligh EG , Dyer WJ (1959) A rapid method of total lipid extraction and purification. Can. J. Biochem. Physiol. 37, 911–917. 13671378 10.1139/o59-099

[acel12491-bib-0010] Boon J , Hoy AJ , Stark R , Brown RD , Meex RC , Henstridge DC , Schenk S , Meikle PJ , Horowitz JF , Kingwell BA , Bruce CR , Watt MJ (2013) Ceramides contained in LDL are elevated in type 2 diabetes and promote inflammation and skeletal muscle insulin resistance. Diabetes 62, 401–410. 23139352 10.2337/db12-0686 PMC3554351

[acel12491-bib-0011] Bui HH , Leohr JK , Kuo MS (2012) Analysis of sphingolipids in extracted human plasma using liquid chromatography electrospray ionization tandem mass spectrometry. Anal. Biochem. 423, 187–194. 22369892 10.1016/j.ab.2012.01.027

[acel12491-bib-0012] Chaurasia B , Summers SA (2015) Ceramides ‐ lipotoxic inducers of metabolic disorders. Trends Endocrinol. Metab. 26, 538–550. 26412155 10.1016/j.tem.2015.07.006

[acel12491-bib-0013] Chiu HC , Kovacs A , Ford DA , Hsu FF , Garcia R , Herrero P , Saffitz JE , Schaffer JE (2001) A novel mouse model of lipotoxic cardiomyopathy. J. Clin. Invest. 107, 813–822. 11285300 10.1172/JCI10947 PMC199569

[acel12491-bib-0014] Coen PM , Goodpaster BH (2012) Role of intramyocellular lipids in human health. Trends Endocrinol. Metab. 23, 391–398. 22721584 10.1016/j.tem.2012.05.009 PMC4908975

[acel12491-bib-0015] Coen PM , Dubé JJ , Amati F , Stefanovic‐Racic M , Ferrell RE , Toledo FG , Goodpaster BH (2010) Insulin resistance is associated with higher intramyocellular triglycerides in type I but not type II myocytes concomitant with higher ceramide content. Diabetes 59, 80–88. 19833891 10.2337/db09-0988 PMC2797948

[acel12491-bib-0016] Ekelund LG , Haskell WL , Johnson JL , Whaley FS , Criqui MH , Sheps DS (1988) Physical fitness as a predictor of cardiovascular mortality in asymptomatic North American men. The Lipid Research Clinics Mortality Follow‐up Study. N. Engl. J. Med. 319, 1379–1384. 3185648 10.1056/NEJM198811243192104

[acel12491-bib-0017] Fleg JL , Morrell CH , Bos AG , Brant LJ , Talbot LA , Wright JG , Lakatta EG (2005) Accelerated longitudinal decline of aerobic capacity in healthy older adults. Circulation 112, 674–682. 16043637 10.1161/CIRCULATIONAHA.105.545459

[acel12491-bib-0018] Gonzalez‐Covarrubias V (2013) Lipidomics in longevity and healthy aging. Biogerontology 14, 663–672. 23948799 10.1007/s10522-013-9450-7

[acel12491-bib-0019] Hammad SM , Pierce JS , Soodavar F , Smith KJ , Al Gadban MM , Rembiesa B , Klein RL , Hannun YA , Bielawski J , Bielawska A (2010) Blood sphingolipidomics in healthy humans: impact of sample collection methodology. J. Lipid Res. 51, 3074–3087. 20660127 10.1194/jlr.D008532 PMC2936747

[acel12491-bib-0020] Haughey NJ , Cutler RG , Tamara A , McArthur JC , Vargas DL , Pardo CA , Turchan J , Nath A , Mattson MP (2004) Perturbation of sphingolipid metabolism and ceramide production in HIV‐dementia. Ann. Neurol. 55, 257–267. 14755730 10.1002/ana.10828

[acel12491-bib-0021] Haus JM , Kashyap SR , Kasumov T , Zhang R , Kelly KR , Defronzo RA , Kirwan JP (2009) Plasma ceramides are elevated in obese subjects with type 2 diabetes and correlate with the severity of insulin resistance. Diabetes 58, 337–343. 19008343 10.2337/db08-1228 PMC2628606

[acel12491-bib-0022] Hawkins MN , Raven PB , Snell PG , Stray‐Gundersen J , Levine BD (2007) Maximal oxygen uptake as a parametric measure of cardiorespiratory capacity. Med. Sci. Sports Exerc. 39, 103–107. 17218891 10.1249/01.mss.0000241641.75101.64

[acel12491-bib-0023] Hill AV , Lupton H (1923) Muscular exercise, lactic acid, and the supply and utilization of oxygen. QJM os‐16, 135–171.

[acel12491-bib-0024] Huang H , Kasumov T , Gatmaitan P , Heneghan HM , Kashyap SR , Schauer PR , Brethauer SA , Kirwan JP (2011) Gastric bypass surgery reduces plasma ceramide subspecies and improves insulin sensitivity in severely obese patients. Obesity (Silver Spring). 19, 2235–2240. 21546935 10.1038/oby.2011.107 PMC3809956

[acel12491-bib-0025] Ichi I , Nakahara K , Miyashita Y , Hidaka A , Kutsukake S , Inoue K , Maruyama T , Miwa Y , Harada‐Shiba M , Tsushima M , Kojo S , Kisei Cohort Study Group (2006) Association of ceramides in human plasma with risk factors of atherosclerosis. Lipids 41, 859–863. 17152923 10.1007/s11745-006-5041-6

[acel12491-bib-0026] Ishikawa M , Tajima Y , Murayama M , Senoo Y , Maekawa K , Saito Y (2013) Plasma and serum from nonfasting men and women differ in their lipidomic profiles. Biol. Pharm. Bull. 36, 682–685. 23546298 10.1248/bpb.b12-00799

[acel12491-bib-0027] Jiang XC , Paultre F , Pearson TA , Reed RG , Francis CK , Lin M , Berglund L , Tall AR (2000) Plasma sphingomyelin level as a risk factor for coronary artery disease. Arterioscler. Thromb. Vasc. Biol. 20, 2614–2618. 11116061 10.1161/01.atv.20.12.2614

[acel12491-bib-0028] Maceyka M , Spiegel S (2014) Sphingolipid metabolites in inflammatory disease. Nature 510, 58–67. 24899305 10.1038/nature13475 PMC4320971

[acel12491-bib-0029] Mielke MM , Bandaru VV , Han D , An Y , Resnick SM , Ferrucci L , Haughey NJ (2015) Demographic and clinical variables affecting mid‐ to late‐life trajectories of plasma ceramide and dihydroceramide species. Aging Cell. 14, 1014–1023. 26193443 10.1111/acel.12369 PMC4693456

[acel12491-bib-0030] Montero D (2015) The association of cardiorespiratory fitness with endothelial or smooth muscle vasodilator function. Eur. J. Prev. Cardiol. 22, 1200–1211. 25301872 10.1177/2047487314553780

[acel12491-bib-0031] Park TS , Goldberg IJ (2012) Sphingolipids, lipotoxic cardiomyopathy, and cardiac failure. Heart Fail Clin. 8, 633–4115. 22999245 10.1016/j.hfc.2012.06.003 PMC4548923

[acel12491-bib-0032] Park TS , Yamashita H , Blaner WS , Goldberg IJ (2007) Lipids in the heart: a source of fuel and a source of toxins. Curr. Opin. Lipidol. 18, 277–282. Review. 17495601 10.1097/MOL.0b013e32814a57db

[acel12491-bib-0033] Parker BA , Kalasky MJ , Proctor DN (2010) Evidence for sex differences in cardiovascular aging and adaptive responses to physical activity. Eur. J. Appl. Physiol. 110, 235–246. 20480371 10.1007/s00421-010-1506-7 PMC2929283

[acel12491-bib-0034] Samad F , Hester KD , Yang G , Hannun YA , Bielawski J (2006) Altered adipose and plasma sphingolipid metabolism in obesity: a potential mechanism for cardiovascular and metabolic risk. Diabetes 55, 2579–2587. 16936207 10.2337/db06-0330

[acel12491-bib-0035] Siddique MM , Li Y , Chaurasia B , Kaddai VA , Summers SA (2015) Dihydroceramides: from bit players to lead actors. J. Biol. Chem. 290, 15371–15379. 25947377 10.1074/jbc.R115.653204 PMC4505450

[acel12491-bib-0036] Smith ME , Tippetts TS , Brassfield ES , Tucker BJ , Ockey A , Swensen AC , Anthonymuthu TS , Washburn TD , Kane DA , Prince JT , Bikman BT (2013) Mitochondrial fission mediates ceramide‐induced metabolic disruption in skeletal muscle. Biochem. J. 456, 427–439. 24073738 10.1042/BJ20130807

[acel12491-bib-0037] Stone JL , Norris AH (1966) Activities and attitudes of participants in the Baltimore longitudinal study. J. Gerontol. 21, 575–580. 5918312 10.1093/geronj/21.4.575

[acel12491-bib-0038] Symons JD , Abel ED (2013) Lipotoxicity contributes to endothelial dysfunction: a focus on the contribution from ceramide. Rev. Endocr. Metab. Disord. 14, 59–68. 23292334 10.1007/s11154-012-9235-3 PMC4180664

[acel12491-bib-0039] Varlamov O , Bethea CL , Roberts CT Jr (2015) Sex‐specific differences in lipid and glucose metabolism. Front Endocrinol. (Lausanne) 5, 241. 25646091 10.3389/fendo.2014.00241 PMC4298229

[acel12491-bib-0040] Weir JM , Wong G , Barlow CK , Greeve MA , Kowalczyk A , Almasy L , Comuzzie AG , Mahaney MC , Jowett JB , Shaw J , Curran JE , Blangero J , Meikle PJ (2013) Plasma lipid profiling in a large population‐based cohort. J. Lipid Res. 54, 2898–2908. 23868910 10.1194/jlr.P035808 PMC3770102

[acel12491-bib-0041] Xia JY , Holland WL , Kusminski CM , Sun K , Sharma AX , Pearson MJ , Sifuentes AJ , McDonald JG , Gordillo R , Scherer PE (2015) Targeted induction of ceramide degradation leads to improved systemic metabolism and reduced hepatic steatosis. Cell Metab. 22, 266–278. 26190650 10.1016/j.cmet.2015.06.007 PMC4527941

[acel12491-bib-0042] Yagyu H , Chen G , Yokoyama M , Hirata K , Augustus A , Kako Y , Seo T , Hu Y , Lutz EP , Merkel M , Bensadoun A , Homma S , Goldberg IJ (2003) Lipoprotein lipase (LpL) on the surface of cardiomyocytes increases lipid uptake and produces a cardiomyopathy. J. Clin. Invest. 111, 419–426. 12569168 10.1172/JCI16751 PMC151861

[acel12491-bib-0043] Yu J , Pan W , Shi R , Yang T , Li Y , Yu G , Bai Y , Schuchman EH , He X , Zhang G (2015) Ceramide is upregulated and associated with mortality in patients with chronic heart failure. Can. J. Cardiol. 31, 357–363. 25746025 10.1016/j.cjca.2014.12.007

[acel12491-bib-0044] Zhang QJ , Holland WL , Wilson L , Tanner JM , Kearns D , Cahoon JM , Pettey D , Losee J , Duncan B , Gale D , Kowalski CA , Deeter N , Nichols A , Deesing M , Arrant C , Ruan T , Boehme C , McCamey DR , Rou J , Ambal K , Narra KK , Summers SA , Abel ED , Symons JD (2012) Ceramide mediates vascular dysfunction in diet‐induced obesity by PP2A‐mediated dephosphorylation of the eNOS‐Akt complex. Diabetes 61, 1848–1859. 22586587 10.2337/db11-1399 PMC3379648

[acel12491-bib-0045] Zhou YT , Grayburn P , Karim A , Shimabukuro M , Higa M , Baetens D , Orci L , Unger RH (2000) Lipotoxic heart disease in obese rats: implications for human obesity. Proc. Natl Acad. Sci. USA 97, 1784–1789. 10677535 10.1073/pnas.97.4.1784 PMC26513

