# Supplementary figures and images for: Circulating ceramides are inversely associated with cardiorespiratory fitness in participants aged 54–96 years from the Baltimore Longitudinal Study of Aging
Source: Aging Cell. 2016 May 2;15(5):825–31. doi: 10.1111/acel.12491 (PMC5013023; doi:10.1111/acel.12491)

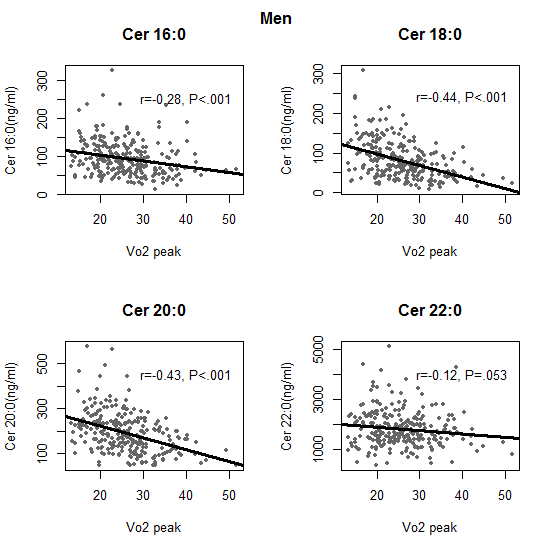

Supplement: Supplementary file 1 — Fig. S1 Plots of ceramide species significantly and inversely correlated with VO2 peak in men. [file ACEL-15-825-s001.tiff]

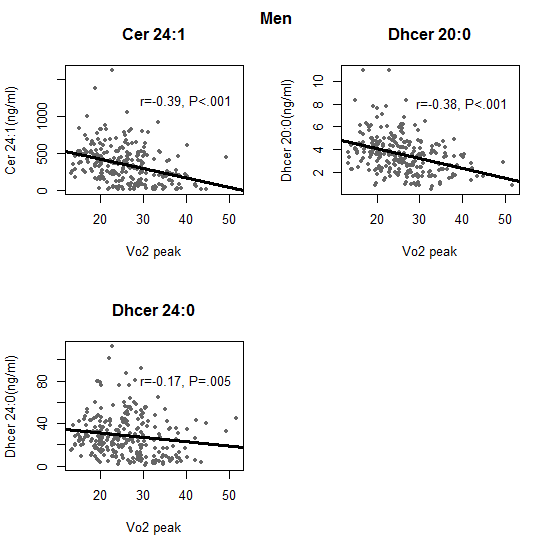

Supplement: Supplementary file 2 [file ACEL-15-825-s002.tiff]

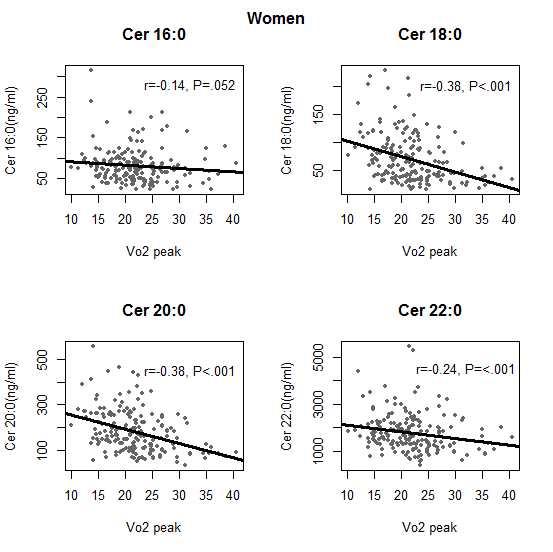

Supplement: Supplementary file 3 — Fig. S2 Plots of ceramide species significantly and inversely correlated with VO2 peak in women. [file ACEL-15-825-s003.tiff]

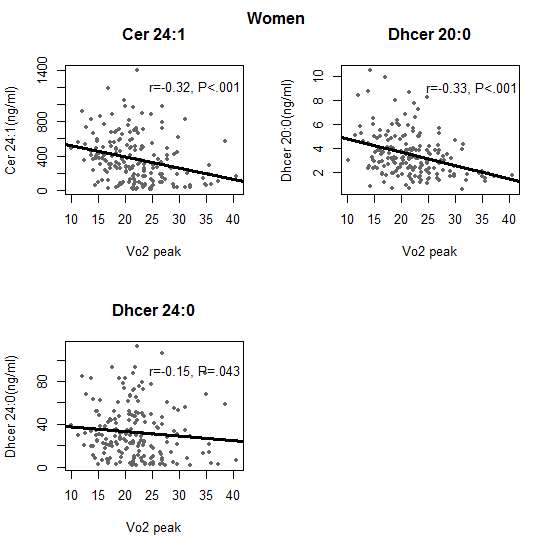

Supplement: Supplementary file 4 [file ACEL-15-825-s004.tiff]
