# Supplementary material for: Circulating ceramides are inversely associated with cardiorespiratory fitness in participants aged 54–96 years from the Baltimore Longitudinal Study of Aging
Source: Aging Cell. 2016 May 2;15(5):825–31. doi: 10.1111/acel.12491 (PMC5013023; doi:10.1111/acel.12491)
Supplement: Supplementary file 5 — Table S1 Mean and standard deviation (SD) of ceramide species (ng mL−1) according to women and men. TableS2 Main characteristics of the population (mean or percentage), according to sex‐specific tertiles of VO2 peak, and their corresponding P trends. Table S3 Spearman correlations exploring the relationship between sphingomyelins and VO2 peak, independent of potential confounders. [file ACEL-15-825-s005.docx]

**Supplemental Materials**

**TableS1.** Mean and standard deviation (SD) of ceramide species (ng/ml) according to women and men.

| **Gender** | **Lipid Species** | N | Mean | Standard deviation (SD) |
| --- | --- | --- | --- | --- |
| **Women** | \| **Total**  **ceramides** \| \| --- \| \| **Cer 16:0** \| \| **Cer18:0** \| \| **Cer20:0** \| \| **Cer22:0** \| \| **Cer24:0** \| \| **Cer26:0** \| \| **Cer22:1** \| \| **Cer24:1** \| \| **Dhcer20:0** \| \| **Dhcer24:0** \| \|  \| | \| 185 \| \| --- \| \| 185 \| \| 185 \| \| 185 \| \| 185 \| \| 185 \| \| 185 \| \| 185 \| \| 185 \| \| 185 \| \| 185 \| | \| 9824.5 \| \| --- \| \| 81.1 \| \| 69.1 \| \| 180.7 \| \| 1778.4 \| \| 7208.4 \| \| 118.0 \| \| 23.2 \| \| 365.6 \| \| 3.4 \| \| 32.5 \| | \| 4160.4 \| \| --- \| \| 42.7 \| \| 43.2 \| \| 94.6 \| \| 793.6 \| \| 3245.6 \| \| 76.4 \| \| 13.4 \| \| 270.6 \| \| 1.8 \| \| 24.5 \| |
| **Men** | \| **Total ceramides** \| \| --- \| \| **Rcer160** \| \| **Rcer180** \| \| **Rcer200** \| \| **Rcer220** \| \| **Rcer240** \| \| **Rcer260** \| \| **Rcer221** \| \| **Rcer241** \| \| **Rdhcer200** \| \| **Rdhcer240** \| | \| 258 \| \| --- \| \| 258 \| \| 258 \| \| 258 \| \| 258 \| \| 258 \| \| 258 \| \| 258 \| \| 258 \| \| 258 \| \| 258 \| | \| 10073.1 \| \| --- \| \| 92.9 \| \| 80.7 \| \| 190.9 \| \| 1828.1 \| \| 7395.2 \| \| 117.7 \| \| 21.9 \| \| 345.5 \| \| 3.5 \| \| 28.3 \| | \| 4122.0 \| \| --- \| \| 45.1 \| \| 47.2 \| \| 89.2 \| \| 729.4 \| \| 3351.3 \| \| 71.3 \| \| 12.9 \| \| 246.3 \| \| 1.7 \| \| 19.7 \| |

**TableS2.** Main characteristics of the population (mean or percentage), according to sex-specific tertiles of VO_2_ peak, and their corresponding P trends.

|  | **VO_2_ peak** | | |  |
| --- | --- | --- | --- | --- |
|  | **Low (n=147)** | **Medium (n=148)** | **High (n=148)** | **P trends** |
| Age, years | 74.7 | 68.3 | 63.7 | <.001 |
| Sex (men) | 58.5% | 58.1% | 58.1% | .900 |
| Race (white) | 78.9 % | 79.0 % | 89.9 % | .337 |
| Height, cm | 168.2 | 169.9 | 170.2 | .290 |
| Weight, kg | 78.8 | 76.3 | 72.5 | .005 |
| Systolic blood pressure, mmHg | 131.6 | 130.1 | 124.8 | .008 |
| Diastolic blood pressure, mmHg | 75.0 | 75.6 | 75.8 | .816 |
| Cholesterol LDL, mg/dl | 114.9 | 115.9 | 116.6 | .967 |
| Cholesterol HDL, mg/dl | 49.7 | 51.2 | 53.1 | .181 |
| Triglycerides, mg/dl | 113.0 | 115.1 | 110.9 | .841 |
| Smokers (current or former), % | 61.2 % | 60.8 % | 58.8 % | .133 |
| Diabetes, % | 10.9 % | 5.4 % | 2.0 % | .032 |
| Pre-diabetes, % | 27.2 % | 34.5 % | 33.8 % | .800 |
| Levels of physical activity, %   - Very low - Low | 10.2%  43.5% | 4.7%  38.5 % | 4.0%  29.7% | .023  .007 |

**Table S3** Spearman correlations exploring the relationship between sphingomyelins and VO_2_ peak, independent of potential confounders

|  | **VO_2_ peak** | | | |
| --- | --- | --- | --- | --- |
| **Lipid species** | **Correlation coefficient** | **P value** | **Bonferroni correction** | **BH correction** |
| Total Sphingomyelins | 0.02 | .629 |  |  |
| Sphingomyelin C16:0 | 0.03 | .544 | 1 | .846 |
| Sphingomyelin C18:0 | -0.07 | .136 | 1 | .470 |
| Sphingomyelin C20:0 | 0.09 | .072 | 1 | .336 |
| Sphingomyelin C22:0 | 0.05 | .325 | 1 | .569 |
| Sphingomyelin C24:0 | 0.09 | .044 | .616 | .308 |
| Sphingomyelin C16:1 | 0.07 | .168 | 1 | .470 |
| Sphingomyelin C18:1 | -0.06 | .208 | 1 | .485 |
| Sphingomyelin C20:1 | 0.01 | .797 | 1 | .929 |
| Sphingomyelin C22:1 | 0.002 | .965 | 1 | .965 |
| Sphingomyelin C24:1 | 0.02 | .646 | 1 | .904 |
| Dihydrosphingomyelin C16:0 | 0.01 | .905 | 1 | .965 |
| Dihydrosphingomyelin C18:0 | -0.01 | .790 | 1 | .929 |
| Dihydrosphingomyelin C22:0 | 0.05 | .324 | 1 | .569 |
| Dihydrosphingomyelin C24:0 | 0.11 | .032 | .448 | .308 |
